# The future of cataract surgery

**DOI:** 10.1038/s41433-025-03745-x

**Published:** 2025-03-13

**Authors:** David O’Brart

**Affiliations:** 1 Department of Ophthalmology, St. Thomas’ Hospital, Lambeth Palace Road, London, SE1 7EH UK; 2 King’s College, London, WC2R 2LS UK

**Keywords:** Eye diseases, Health occupations, Drug discovery

## Abstract

The topic of the 2024 Cambridge Ophthalmology Symposium was “Evolution and the Eye”. The topic of this paper is to discuss various “evolutionary” pressures that may shape the future of cataract surgery (CS) over the next decades. These pressures include: The need to improve CS access; The need to improve sustainability; The development and introduction of new technologies, and the incorporation of artificial intelligence.

## Introduction

Cataract surgery (CS) is one of the most cost-effective and commonest surgeries undertaken [1] with approximately 3.8 million performed annually in the USA, above 4.3 million in Europe and over 20 million internationally [2, 3]. The last seventy years have seen great advances. The development of modern micro-incisional techniques and intraocular lenses (IOLs) are such that visual outcomes can be excellent, with the UK’s National Ophthalmic Database study reporting over 80% of eyes, without co-pathology achieving 6/12 or better uncorrected acuity [4, 5]. Thus, CS is one of the most successful interventions in modern medicine.

It is difficult to predict what the future holds for CS over the next seventy years in today’s uncertain world. It is the author’s belief that certain “evolutionary” pressures will shape its future, including: The need to improve CS access; The need to improve sustainability; The development and introduction of new technologies, and the incorporation of artificial intelligence (AI).

## The need to improve access to Cataract Surgery

Cataract is the main cause of global blindness (bilateral acuity <3/60), with approximately 15.2 million cases (45% of blindness), and the second cause of moderate-to-severe visual impairment (MSVI) (bilateral acuity <6/18), with 78.8 million cases (39% of MSVI) [6–8]. Compared to high income countries (HICs), low/middle-income countries (LMICs) are disproportionately affected, with blindness/MSVI rates in sub-Saharan Africa (SSA) and Southeast Asia eight times higher than in HICs [9]. The most vulnerable (the poor, the elderly, the illiterate, women, ethnic minorities, rural and indigenous populations) are disproportionally affected with less access to quality surgery [10].

Despite efforts internationally to reduce poverty, disease burden, and improve access to eye care, the numbers who are blind/have MSVI, including those with cataract, are predicted to increase, from 338.3 million in 2020 to 535 million in 2050 due to global population growth, population aging, and increasing rates of diseases associated with blindness/MSVI. [9, 11]. The global financial burden of visual loss is estimated at $441 billion USD [1], while the costs of addressing the coverage gap for cataract blindness/MSVI are $8.8 billion USD [11]. It makes both economic and moral sense that one of the future paths of CS is directed at reducing global cataract blindness/MSVI.

There are barriers to addressing cataract blindness/MSVI in LMICs, including poor awareness of services, unaffordable user fees/transport costs, and limited numbers of eye care facilities and eye care professionals (ECPs), especially in rural areas [12]. These challenges should not be insurmountable and may be overcome by investment in and development of models of integrated people-centred eye care. For example, the Arvind model in India [13, 14] delivers efficient, high-quality, high-volume CS using outreach clinics, providing patient transportation and cross-subsidisation from higher income patients to allow free/subsidised costs for low-income patients [13, 14]. Their implementation, in regions such as SSA may be problematic due to poor infrastructure, paucity of ECPs and more widely dispersed populations [14]. However, similar models are beginning to emerge in these areas [15] and it might be expected that advances in surgical simulation training, telemedicine, artificial intelligence (AI) and digital imaging can improve ECP training and overcome geographical barriers, allowing underserved populations access to eye care.

It is unnecessary to invest in expensive and complex CS technologies in LMICs. With manual small incision CS (MSICS), the cost of a surgery can be as low as $20 USD, with operating times less than 10 minutes, allowing high volume, sustainable, cost-effective models of delivery [16, 17]. Outcomes are good. One study comparing MSICS with phacoemulsification reported 85% had uncorrected acuities of 6/18 or better post-MSICS [18], with shorter surgical times and costs, with a Cochrane review finding similar post-operative corrected acuities compared to phacoemulsification CS (PCS) and low complication rates [19].

There is a paucity of cataract surgeons in LMICs, especially in SSA, where there are 2.5 Ophthalmologists per million inhabitants compared to 76.2 in HICs [20, 21]. Reasons include limited training institutions, facilities and funding [21, 22], compounded by a “brain-drain” as ECPs are enticed to work abroad by higher salaries, superior education/career progression and more supported and better working conditions [23]. There is a future need to invest in Ophthalmology training programmes in LMICs to provide sustainable conduits of qualified ECPs, conceivably subsidised by HICs to offset the effects of the brain drain which benefits HICs’ healthcare systems to the detriment of those in LMICs. To retain Ophthalmologists, competitive salaries, better education and career opportunities need to be offered in LMICs, with investment in eye care to provide better structured and equipped working environments. One response to Ophthalmologist insufficiency in SSA since the mid-1980s has been the training of clinical officers and ophthalmic nurses to become non-physician cataract surgeons (NPCSs) [24, 25], to facilitate shorter time periods of training [24], and easier retention and deployment in remote areas [25]. Retention of NPCSs is generally good, but productivity limited as NPCSs often work in district hospitals with few ECPs to support them and constrained equipment and supplies [26]. However, NPCS productivity as high as 1500 cases per year is reported in some settings, suggesting that with the correct infrastructure, high volume models can be achieved [26]. Published outcome data is limited. One study reported outcomes for NPCS performing extracapsular CS comparable to Ophthalmologists performing similar CS in LMICs [27], but further studies are necessary to ensure quality care. Regrettably, there is global inequality in outcomes with a systematic review reporting that in LMICs, with surgery performed by Ophthalmologists, outcomes were typically below 70% for presenting acuities of 6/18 or better [28]. It appears that as well as a future need to increase cataract surgeon numbers in LMICs, these surgeons need quality training, especially in MSICS techniques given its efficacy/cost-efficacy [16–19], with access to continuing medical education, and require support in relation to other ECPs, working infrastructure and equipment, to ensure outcomes reach WHO recommendations of 80% with presenting acuities of 6/18 or better [29].

Demands for CS are increasing in HICs, due to population growth/aging, increased rates of cataract-associated disease [30], CS conducted at earlier stages [31] and backlogs caused by the COVID-19 pandemic [32]. This demand necessitates improvement in productivity. This is pertinent to the National Health Service (NHS) where problems with CS productivity have been reported [33]. Using time-and-motion studies to identify staffing levels and key tasks performed by ECPs to optimise surgical productivity in NHS settings [34–36], we found that the numbers of non-ophthalmologist ECPs supporting surgeons, the number of key tasks performed, and time taken to perform these tasks were predictors of the time taken to perform CS and the time spent by patients in the operating theatre (OT) [34]. This allowed us to highlight models of CS delivery with up to 14 cases per 4-hour list [34] and more if immediate sequential bilateral cataract surgery (ISBCS) was undertaken [35, 36]. NHS units that had the highest productivity had the longest duration of staff breaks, indicating that they were working “smarter not harder”, supporting surgeons so they were operating, not undertaking other tasks [34].

While NHS CS numbers have increased to meet demand, with almost 500,000 cases in England and Wales in 2021 [37], this has been driven by the independent sector. Prior to the COVID-19 pandemic the percentage of NHS CS performed by the independent sector never exceeded 30%. It is now 60%, with a concurrent reduction by NHS providers [38]. This has raised concerns about meeting NHS CS training requirements, deskilling of NHS Ophthalmologists, underfilling of CS lists, loss of ECPs to the independent sector and financial instability of NHS Ophthalmology units with reduced ability to treat other eye conditions [39]. In the author’s unit there has been loss of a fellowship post, high volume ISBCS lists and reduced recruitment to cataract research because of such issues. If the NHS could improve future CS productivity in line with the high-volume delivery models discussed in our studies [34–36], it probably has enough theatre capacity and surgeons to meet current demands. Why this is not happening is contentious, but disconnects in management, entrenched working practices, lack of investment and old infrastructure may be contributory factors. Future autonomy of NHS ophthalmic units may be necessary to overcome such obstacles and deliver productivity to meet CS demands without reliance on the independent sector.

## The need to improve sustainability in Cataract Surgery

Climate change from greenhouse gases (GHG) is threatening global health [39]. Natural disasters, food and water insecurity, population migration and infectious disease risks are predicted to present challenges in coming decades [39]. Healthcare is a large contributor to GHG emissions. The NHS accounts for 4.6% of the UK’s carbon footprint (CF) [40]. Surgery has a disproportionate impact because of energy-intensive processes, resource consumption and waste production [41]. Given its frequency, the reduction of CS-associated CFs are important.

In HICs the CF of phacoemulsification CS ranges from 78.4 to 181.98 kilograms (Kg) CO^2^ equivalents [42–47]. In one study, procurement, including supply chains for equipment (33%), pharmaceuticals (18%) and waste management (2%), were responsible for over half the CF, with the remainder due to building and energy use (36%) and travel (patients 7%, staff 3%) [42]. At Aravind Eye hospital, the CF is 6Kg CO^2^ equivalents [48], achieved by high volume models and re-using surgical equipment/pharmaceuticals (not changing surgical gowns and gloves, re-using phacoemulsification tips, cassettes, tubing, metal blades, cannulas, sutures and using perioperative medications for multiple patients) [48]. Their reported endophthalmitis rate using intracameral moxifloxacin is only 0.01% [49]. This suggests that some mandated practices in HICs, responsible for high CFs in CS, might be unnecessary. Given the CF contribution of medical equipment/pharmaceuticals, research into strategies for the future safe and sustainable re-use of surgical instruments, phacoemulsification cassettes/tubing/tips, and more sustainable strategies for pharmaceuticals is indicated. Recent studies have detected no ultrastructural damage after repeated use of phacoemulsification tips [50] and demonstrated efficacy with reusable phacoemulsification cassettes [51] and drop-less cataract surgery [52]. It might be also hoped that future AI-driven supply chains will reduce GHG emissions, by optimising processes related to manufacture, distribution and procurement of CS equipment/pharmaceuticals [53].

IOL packaging weights range from 29 to 80 grams (g), while the lens is only 1g [54]. Most of this is attributable to the instructions for use pamphlet. Providing this electronically reduces CF and has been undertaken by some manufacturers [55]. Hopefully this and other strategies to minimise packaging will become the norm for all CS equipment/pharmaceuticals. Software tools to improve sustainability of CS packs are available [56]. The “sustainability index for disposables in cataract surgery” software developed by the European Society of Cataract and Refractive Surgeons allows packs to be compared with their benchmark recommendations and highlights equipment changes that can be made to reduce CFs [56].

To limit the CF of building and energy use, maximising future CS productivity is essential [34–36]. Simple measures such as occupancy sensors to turn off lights in unoccupied rooms, using diode lighting and limiting air changes to 25 per hour [57], but starting these only 30 minutes before commencement of CS, are helpful.

Travel-associated CFs can be reduced by performing ISBCS routinely, which improves surgical productivity [35, 36] and reduces patient travel/appointments, and by providing pre- and post-operative appointments in the community. At the author’s unit, and many others, these are provided by community-based accredited Optometrists. This is likely to be the future norm.

Importantly, the strategies, outlined above, to improve sustainability are also cost saving, which will hopefully help the drive towards greener CS.

## The development and introduction of new technologies

### Robotics

In CS there is direct visualization of the surgical workspace, unrestricted instrument manoeuvrability and small incision technologies, allowing manual techniques to afford excellent outcomes [4, 5]. However, manual surgery will always be restricted by physiological constraints, such as hand tremor and limitations of tactile and depth perception. Future robotics may overcome these limitations. Femtosecond lasers, such as the Keranova system, use laser wavefront modification to create scanning beams with multiple focused spots and shapes and energy threshold mapping throughout the cataract to limit gas formation, allowing cataracts to be dissected into homogenous volumes of 200-µm cubes that can be aspirated without phacoemulsification energy [58]. Such innovations together with robotic platforms, including the Intraocular Robotic Interventional Surgical System (IRISS), which can undertake capsulorhexis and lens cortex removal [59], coupled with real time optical coherence tomography imaging, which with the IRISS enables lens capsule polishing [60] and lens removal [61], herald the possibility of fully automated robotic CS. Robotics with their ultra-precise surgical movements, microscopic real-time imaging and integrated AI might offer a CS future with ultimate patient safety and outcomes, perhaps allowing productivity improvements, with surgeons operating multiple systems at once, and even permit better global CS access.

Its introduction is not without challenges. There will be developmental costs, which need recouping, and once implemented maintenance costs for complex systems will be necessary. The technology will have to be affordable, meet current and future sustainability requirements, offer better outcomes/patient safety and/or allow productivity improvements while remaining cost-effective. Femtosecond laser-assisted CS (FLACS) has not provided such benefits, limiting its usage to the private sector. Compared to PCS, outcomes are similar [62] and we demonstrated that even with models to improve productivity extra FLACS-associated costs cannot be offset [63].

### Multifocal and accommodative IOLs

Advances since the first IOL was implanted 75 years ago [64] are such that CS is not just sight-restoring. There is now a plethora of IOLs with complex optics, including multifocals, designed to provide spectacle-independence for near and far. Whilst recent multifocal lenses report spectacle-independence in over 90% [65], due to their optics they are not recommended in eyes with compromised vision from other comorbidities and are associated with reduced contrast and dysphotopsias (halos/starbursts), which in a minority necessitates IOL exchange [66]. New models, such a recent AI-created non-diffractive spiral design may limit dysphotopsias while providing full vision ranges [67], but the “holy grail” is to reproduce the physiology of accommodation. If developed, such accommodative IOLs might allow spectacle-independence without contrast loss and minimal dysphotopsias. Designs have been tested over past two decades with limited success [68], but new models offer promise. One design type is inserted to fill the capsular bag and comprise a fluid lens, with or without a secondary refractive lens, that changes shape with tensions transmitted to the capsular bag from the ciliary muscle. Examples are the Juvene [69], the Fluidvision [70], and the OmniVu lenses [71], with initial studies indicating amplitudes of accommodation from 2.2 to 4.0 dioptres [69–71]. The second type is placed in the ciliary sulcus and relies on direct ciliary body contraction to achieve an accommodative effect. Examples are the Lumina lens [72], which is CE marked and undergoing multicentre trials and the Opira lens. Clinical trials over the next years will elucidate the efficacy and biocompatibility of these lenses and may herald a future of truly accommodative IOLs.

### Drops instead of surgery

There are no approved drops to treat cataracts, but several compounds are under investigation and may offer future promise. Sterols such as Lanosterol have been purported to be able to help lessen protein aggregates present in cataracts [73], but laboratory results have been mixed [74]. Lanosterol is a large molecule, limiting its topical delivery. 5-cholesten-3b,25-diol (VP1-001), is more soluble and has been shown to clear cataracts in mice [75]. Antioxidants such as Rosmarinic acid and N-acetylcarnosine have been reported to improve lens opacity in cataract models [76, 77]. There are a few limited clinical studies using N-acetylcarnosine with over the counter drop available, but further evidence is required to support efficacy [77, 78].

## Artificial intelligence and cataract surgery

Increased computational power and large data sets have facilitated AI proliferation, which has been applied to the management of diabetic retinopathy, age-related macular degeneration and glaucoma and is now being implemented in CS [79].

Machine and deep learning algorithms can recognize cataracts pre-operatively based on anterior segment slit-lamp images or fundal image distortions [80, 81]^,^ with systems able to detect corneal co-pathologies [82]. This affords potential technician-led cataract detection and risk stratification even in remote areas in LMICs.

AI-assisted IOL power calculations can incorporate complex nonlinear relationships between ocular parameters. The Ladas Super formula combines existing formulae and a data set of 4000 eyes for deep learning [83]. The Kane formula utilises theoretical optics, thin lens formulae and ‘big data’ [84]. Both are more accurate than 3rd or 4th generation formulae [85]. It is anticipated that future AI-formulae will further improve refractive outcomes.

Intraoperatively, AI-based systems for image and video analysis allow CS phase recognition, CS instrument detection and movement [86, 87], offering future promise for AI-driven CS context-aware assistance, surgical monitoring, duration prediction, phase detection, and intraoperative risk stratification to enhance safety and efficiency [88].

AI offers the development of “Smart Theatres” with video management systems, improved connectivity, integration between complex technologies, surgical device/robotics control and instrument tracking and procurement amongst other features [89]. Recently, proof of concept of an eye-tracking robotic scrub nurse has been reported to give the surgical team a “third hand” in the future [90].

Surgical simulators have been shown to accelerate skills transfer and improve safety [91]. They are valuable tools for surgical training. In the future they will probably be incorporated for all surgeon grades for training new procedures, revalidation and return to work after a hiatus to avoid deskilling [92, 93], as in the aviation industry. AI can discriminate resident from senior surgeons by evaluating instrument pathlength, movement and time [87] and stratify performance. In the future, AI-driven simulators may offer feedback, develop normative pools and correlate with clinical outcomes.

AI-driven post-op data set integration with intra- and pre-operative data, should allow refined complication and outcome analysis, affording better outcomes and tailored healthcare, for example deciding which “premium” IOL is best suited to an individual patient. AI systems also have been applied to detect posterior capsular opacification and could help to triage patients for laser capsulotomy [94] and future better data integration between primary and secondary healthcare should improve patient scheduling [95].

## Conclusion

CS is a highly successful medical intervention. Technological innovations and incorporation of AI should ensure a bright future. Improving sustainability and increasing future access are challenges to overcome.
